# Stress perfusion cardiac MRI in women

**DOI:** 10.1186/1532-429X-13-S1-P87

**Published:** 2011-02-02

**Authors:** Darach O h-Ici, Thierry Unterseeh, Thomas Hovasse, Yves Louvard, Marie Claude Morice, Jérôme Garot

**Affiliations:** 1 Institut Cardiovasculaire Paris Sud, Massy, France

## Background

Cardiac perfusion and stress MRI appear to represent a promising approach for detecting coronary artery disease. It provides valuable information that may not be available from other diagnostic tools such as echocardiography and nuclear cardiology. A recent consensus panel has assigned a Class-II recommendation for the use of myocardial perfusion imaging for the detection of coronary artery disease. However limited results are available for the use of these technologies in women. The aim of this study was to describe the findings in women and compare them to men.

## Methods

All patients who underwent stress CMR at ICPS (tests, n = 4589) between November 2009 and September 2010 were identified through the use of the cardiology database. For patients who underwent multiple tests during this time period (n = 116), only the first test was included. The study population consisted of 4584 patients.

## Results

Women had a similar number of risk factors to Men (2.3 ± 1.2, p = ns), but were significantly older (67.4 vs 64 yrs, p<0.001). They were also more likely to have diabetes (28.7% vs 25.4%, p=0.02) but less likely to smoke (17.2% vs 30.9%, p <0.001). In relation to previous cardiac history, women were less likely to have had PCI (19.1& vs 33.3%, p < 0.001), coronary artery bypass grafting, or myocardial infarction ((7.1& vs 16.3% , p< 0.001).

Women have less ischemia (12.42% vs 21.79, p<0.001) but a similar likelihood of infarct (25.20% vs 23.74%, p = 0.29). Asymptomatic women had less ischemia than asymptomatic male (12.0% vs 21.5%, p<0.001). Symptomatic women had less ischemia than asymptomatic Men (13.0% vs 21.45%, p<0.001). Asymptomatic and symptomatic women have similar rates of ischemia (12.0% vs 13.0%, p = 0.575).

## Conclusion

In spite of a similar risk profile to men, they have much less ischemia on testing. The incidence of myocardial infarction is similar in both men and women in this population suspected of coronary artery disease. The presence of symptoms, either chest pain or shortness of breath, does not increase the likelihood of ischemia in women. Myocardial infarction not related to epicardial artery stenoses may play a role in explaining the presence of chest pain in women with normal coronary arteries.

